# Towards a Semantic Lexicon for Biological Language Processing

**DOI:** 10.1002/cfg.451

**Published:** 2005

**Authors:** Karin Verspoor

**Affiliations:** Los Alamos National Laboratory PO Box 1663 MS B256 Los Alamos NM 87545 USA

## Abstract

This paper explores the use of the resources in the National Library of Medicine's Unified Medical Language System (UMLS) for the construction of a lexicon useful for processing texts in the field of molecular biology. A lexicon is constructed from overlapping terms in the UMLS SPECIALIST lexicon and the UMLS Metathesaurus to obtain both morphosyntactic and semantic information for terms, and the coverage of a domain corpus is assessed. Over 77% of tokens in the domain corpus are found in the constructed lexicon, validating the lexicon's coverage of the most frequent terms in the domain and indicating that the constructed lexicon is potentially an important resource for biological text processing.


**Comparative and Functional Genomics**
*Comp Funct Genom* 2005; **6**: 61–66.
Published online in Wiley InterScience (www.interscience.wiley.com). **DOI:** 10.1002/cfg.451

**Conference Paper**

### Towards a semantic lexicon for biological language processing

Karin Verspoor*
*Los Alamos National Laboratory, PO Box 1663, MS B256, Los Alamos, NM 87545, USA*

**Correspondence to: Karin Verspoor, Los Alamos National Laboratory, PO Box 1663, MS B256, Los Alamos, NM 87545, USA. E-mail: verspoor@lanl.gov*

Received: 9 December 2004
Accepted: 14 December 2004

### Abstract

**This paper explores the use of the resources in the National Library of Medicine's Unified Medical Language System (UMLS) for the construction of a lexicon useful for processing texts in the field of molecular biology. A lexicon is constructed from overlapping terms in the UMLS SPECIALIST lexicon and the UMLS Metathesaurus to obtain both morphosyntactic and semantic information for terms, and the coverage of a domain corpus is assessed. Over 77% of tokens in the domain corpus are found in the constructed lexicon, validating the lexicon's coverage of the most frequent terms in the domain and indicating that the constructed lexicon is potentially an important resource for biological text processing. Copyright © 2005 John Wiley & Sons, Ltd.**

**Keywords:** **natural language processing; lexicon; unified medical language system**

### Introduction

It is well understood that natural language processing (NLP) applications require sophisticated lexical resources to support their processing goals. In the biomedical domain, we are privileged to have access to extensive terminological resources in the form of controlled vocabularies and ontologies, which have been integrated into the framework of the National Library of Medicine's Unified Medical Language System's (UMLS) Metathesaurus. However, the existence of such terminological resources does not guarantee their utility for NLP. In particular, we have two core requirements for lexical resources for NLP in addition to the basic enumeration of important domain terms: representation of morphosyntactic information about those terms, specifically part of speech information and inflectional patterns to support parsing and lemma assignment, and representation of semantic information indicating general categorical information about terms and significant relations between terms, to support text understanding and inference (Hahn *et al.*, 1999). Biomedical vocabularies by and large commonly leave out morphosyntactic information, and where they address semantic considerations, they often do so in an unprincipled manner, e.g. by indicating a relation between two concepts without indicating the type of that relation.

But all is not lost. The UMLS knowledge sources include two additional resources which are relevant — the SPECIALIST lexicon, a lexicon addressing our morphosyntactic requirements, and the Semantic Network, a representation of core conceptual categories in the biomedical domain. The coverage of these two knowledge sources with respect to the full coverage of the Metathesaurus is, however, not entirely clear. Furthermore, when our goals are specifically to process biological text — and often more specifically, text in the molecular biology domain — it is difficult to say whether the coverage of these resources is meaningful. The utility of the UMLS knowledge sources for medical language processing (MLP) has been explored (Johnson, 1999; Friedman *et al.*, 2001); the time has now come to repeat these experiments with respect to biological language processing (BLP). To that end, this paper presents an analysis of the UMLS resources, specifically with an eye towards constructing lexical resources suitable for BLP. We follow the paradigm presented

Copyright © 2005 John Wiley & Sons, Ltd.

in Johnson (1999) for medical language, exploring overlap between the UMLS Metathesaurus and SPECIALIST lexicon to construct a morphosyntactic and semantically-specified lexicon, and then further explore the overlap with a relevant domain corpus for molecular biology.

### The UMLS as a lexical knowledge source

There have been several investigations of the UMLS as a lexical knowledge source. McCray *et al.* (2001) evaluated the nature of strings in the UMLS Metathesaurus with respect to their likelihood of appearing in a natural language corpus. They found that only 10% of the strings in the Metathesaurus occurred in their MEDLINE corpus (representing 1 year of MEDLINE abstracts), but were able to identify some properties associated with the strings that could be used to filter out strings that are unlikely to occur naturally in a corpus. While the authors suggest that occurrence of a term in the Metathesaurus opens the possibility of accessing more extensive domain knowledge about that term, they do not explore the nature of that domain knowledge for the terms they find in their corpus, and do not explore the overlap of those terms with other UMLS resources.

Friedman *et al.* (2001) quantitatively compare a lexicon developed manually for their MEDLEE system with a lexicon derived automatically from the UMLS, with respect to the task of processing clinical information in patient reports. They found the UMLS-derived lexicon led to poor performance relative to their own lexicon. The results do not, however, invalidate the UMLS as an important source of lexical information, as they may simply be a reflection of the completeness of the existing MEDLEE lexicon for the task evaluated. The authors argue that using the UMLS can substantially reduce the manual effort in constructing a lexicon.

Johnson (1999) explores the construction of a lexical resource from the UMLS in support of processing of medical narrative, specifically utilizing a corpus of discharge summaries from hospital visits. Johnson explores the overlap between the Metathesaurus, the SPECIALIST lexicon, and a domain corpus, and presents some strategies for handling semantic ambiguities that arise during the mapping of terms in the different UMLS resources. Johnson found that while 79% of the distinct lexical forms in his corpus occurred in the SPECIALIST lexicon, only 38% of those forms occurred in the semantic lexicon of more than 75 000 entries derived from intersecting the Metathesaurus and the SPECIALIST lexicon — so only 38% of terms in the corpus could be expected to have both morphosyntactic and semantic information derived from the UMLS. Johnson points out this may reflect the fact that the Metathesaurus may contain many complex medical terms that should not be considered lexical items, or that may successfully be incorporated into the lexicon by assuming that they are nouns.

### Methods

We follow Johnson (1999) and explore the overlap in the UMLS Metathesaurus and the SPECIALIST lexicon to establish a baseline semantic lexicon, and then investigate its relevance for a corpus in the molecular biology domain. We utilize the 2003AC UMLS release. As our domain corpus, we utilize 28 874 full text articles from the *Journal of Biological Chemistry* (JBC), spanning the years 1998–2002, originally obtained for the 2003 BioCreAtIvE competition (**http://www.mitre.org/public/biocreative/**). While we realize that this is not a sample representative of the full domain of molecular biology, it is representative of a significant portion of that domain, and the results on JBC texts should be indicative of the coverage of our semantic lexicon for this domain. We felt it preferable to use a corpus of full text articles rather than a corpus of abstracts derived from MEDLINE in order to more completely assess coverage of the relevant language.

The steps for building and evaluating our semantic lexicon are as follows:

- Lexemes in the SPECIALIST lexicon are matched to terms in the Metathesaurus. We load in all the strings represented in the SPECIALIST LRAGR file, and attempt to match Metathesaurus strings extracted from the MRCON file to these strings. This is done by considering different kinds of matches:
  - Exact match.
  - Match after upper-casing the first letter of the SPECIALIST string.

Copyright © 2005 John Wiley & Sons, Ltd. 

- o Match after upper-casing the first letter of each word of the SPECIALIST string.
- o Match after upper-casing the entire SPECIALIST string.
- o Other case-insensitive match.
- o Match (any of the above types) after stripping the Metathesaurus string of ', NOS' or '⟨1⟩', '⟨2⟩', etc. at the end.
- o Finally, consider whether each of the constituent tokens of a multi-token (space containing) Metathesaurus string occurs in the SPECIALIST lexicon (after removal of words consisting of all numbers or punctuation), in order to assume a compositional analysis of the term.

- Filter the resulting lexicon (a subset of the original SPECIALIST lexicon tied to specific Metathesaurus terms) by removing any terms for which the corresponding Metathesaurus string is not associated with a semantic type through one of its associated concepts. There may be concepts for which the UMLS does not provide semantic information, and therefore they do not satisfy our lexical constraints requiring both morphosyntactic and semantic information.
- Search the domain corpus for occurrences of any lexical variant of each term in our semantic lexicon (obtaining lexical variants from the UMLS lexical tools), and track any matches in order to establish the coverage of the lexicon.

### Results

Our results on matching between the SPECIALIST lexicon and the Metathesaurus, shown in Table 1, indicate that the proportion of Metathesaurus terms directly occurring (through some matching paradigm) in the SPECIALIST lexicon is in fact slightly less than Johnson's (1999) finding of 12% at 8.2%. This is due to the incredible growth in the Metathesaurus in the past few years; Johnson reports finding 630 658 unique strings in the Metathesaurus, while the version we worked with contains 1 959 516 unique strings. The SPECIALIST lexicon has grown as well (from 164 850 distinct lexical forms to 292 979), but clearly not at pace with the Metathesaurus. This result is in line with Johnson's observation that many of the terms in the Metathesaurus are probably not appropriate for recording directly in the SPECIALIST lexicon. However, upon inspection of the constituent structure of Metathesaurus terms, we found that for a large proportion of terms (79%), each of the constituent members of the (multi-word) term could be found in the SPECIALIST lexicon. This opens the possibility of a compositional analysis for many Metathesaurus terms, although it does not address the assignment of semantic type to the term as a whole.

**Table 1.** Matches between the UMLS Metathesaurus terms and the SPECIALIST lexicon

| | *n* | (%) |
|---|---|---|
| Exact matches | 58 918 | 3.0 |
| First letter upper case | 67 765 | 3.5 |
| First letter, all words upper case | 13 922 | 0.7 |
| Entire string upper case | 12 961 | 0.7 |
| Other case insensitive match | 1 982 | 0.1 |
| Stripped term matches | 5 945 | 0.3 |
| Total direct matches | 161 493 | 8.2 |
| Constituent matches | 1 548 389 | 79.0 |
| Total matches | 1 709 882 | 87.3 |

The number of unique SPECIALIST terms matched by Metathesaurus terms was 108 295. These string matches were used to create a lexicon containing 96 205 unique entries from the SPECIALIST lexicon (where a given term may correspond to multiple lexical entries due to the morphosyntactic ambiguity of the term, and a given lexical entry may correspond to multiple terms due to lexical variation) by identifying each of the lexical entries to which a matched string may correspond. This is 52% of the complete SPECIALIST lexicon (of 183 301 entries). Filtering this lexicon according to the constraint of having a semantic type for each had no impact whatsoever — we found that each of the 78 595 unique Metathesaurus concepts matched to a SPECIALIST lexicon term was also associated with a semantic type in the Metathesaurus, so there was no reduction in the lexicon. It should be noted that the number of Metathesaurus concept matches is significantly lower than the number of Metathesaurus term matches, because several distinct terms in the Metathesaurus may correspond to the same concept.

We next explored the overlap of the resulting lexicon with our domain corpus, by looking for matches between tokens in the corpus and any lexical form associated with the 96 205 entries in

Copyright © 2005 John Wiley & Sons, Ltd. 

our subset of the SPECIALIST lexicon (whether or not that exact form occurred in the initial Metathesaurus term set). We split each of the 28 874 JBC files into tokens after stripping HTML tags and converting HTML character entities. We investigated several different ways in which a token could match a lexical entry:

- Exact single token match: the token occurs in the lexicon exactly as it appears in the text.
- Case-insensitive single token match: the token in the text matches a lexical entry when matched case insensitively.
- Exact multi-token term match: the token starts a phrase in the text that exactly matches a multi-token term in the lexicon.
- Case-insensitive multi-token term match: the token starts a phrase in the text that matches a multi-token term in the lexicon when matched case insensitively.
- 'Relaxed' hyphenated token match: for single tokens containing hyphens that did not match a lexical entry in some way, we generated a variant of the token with the hyphens replaced by spaces, effectively generating a multi-token term out of the original single token. The following matches were then attempted:
  - Match (exact) of the relaxed token string to a multi-token term in the lexicon.
  - Matching (exact and case-insensitive) where the relaxed token string starts a phrase in the text matching a (longer) multi-token term in the lexicon.
  - If the relaxed token string did not match a multi-token term in the lexicon, attempt to match each of the constituent words of the string to a lexeme.

The results appear in Table 2. We see that over 77% of the tokens in the corpus match exactly to a lexeme in the lexicon, with a total of 83% matching when case-insensitive matches are allowed. Although this corresponds to only approximately 3% of the distinct tokens found in the corpus, the high coverage of the corpus as a whole indicates that this 3% corresponds to the most frequent tokens in the corpus. The lexicon therefore includes the main content-bearing terms of the domain,

**Table 2.** Matches between the derived lexicon and the domain corpus

| | Count | % of base set |
|---|---|---|
| Total number of files processed | 28 874 | |
| Basic token matches | | |
| Number of tokens | 156 608 748 | |
| Single token matches | 121 552 230 | 77.6 |
| Additional matches with case insensitivity | 9 419 429 | 6.0 |
| Multi-token term matches | 2 866 226 | 1.8 |
| Additional multi-token term matches with case insensitivity | 261 128 | 0.2 |
| Number of unique tokens | 1 898 320 | |
| Unique unmatched tokens | 1 836 148 | 97 |
| Unique unmatched numeric tokens | 78 770 | 0.5 |
| Matches for tokens following hyphenation relaxation | | |
| Number of tokens relaxed | 6 869 993 | |
| Number of constituent tokens | 13 994 307 | |
| Relaxed tokens directly matching multi-token term | 157 529 | 2.3 |
| Tokens starting (longer) multi-token term match | 14 100 | 0.2 |
| Additional matches with case insensitivity | 0 | 0.0 |
| Tokens with some constituent match | 4 899 189 | 71.3 |
| Number of constituent tokens matching | 7 396 976 | 52.9 |
| Lexicon matches | | |
| Unique lexemes in lexicon | 292 979 | |
| Unique lexical entry IDs in lexicon | 96 205 | |
| Unique single token lexemes in lexicon | 268 617 | |
| Unique multi-token lexemes in lexicon | 24 362 | |
| Unique single token lexemes matched | 62 172 | 23.1 |
| Unique multi-token lexemes matched | 15 290 | 62.8 |
| Unique lexical entry IDs matched | 59 199 | 61.5 |

Copyright © 2005 John Wiley & Sons, Ltd. 

in addition to the expected grammatical function words such as 'and', 'the', etc.

Inspection of the tokens which did not match any lexeme in the lexicon shows that a large proportion of the unmatched tokens are numeric tokens. This accounts for an additional 7 969 674 of the tokens (5%), though they correspond to only 0.5% of all distinct tokens. Other frequently unmatched token types correspond to chemical formulas (e.g. 'K+'), gene/protein names (e.g. 'ERK2'), typographical errors (e.g. 'negative'), protein sequences (e.g. 'CACAGAGGATGGGTTAACTCCCAG'), proper names, some tokens that only occur as part of a multi-token term (e.g. '*de*' in '*de novo*' or '*vitro*' in '*in vitro*'), as well as many that seem to derive from errors in our tokenization or problems with handling of UNICODE characters. Many of these could be handled by specific tokenization and token-tagging strategies, rather than requiring that the terms be enumerated in the lexicon.

The lexicon does contain a significant number of terms which were not found in the corpus, since only 62% of the lexical entries had a match (on at least one of its lexical variants) in the corpus, but it does not necessarily follow that the remaining 38% of the lexicon is irrelevant for biological language processing, as it could be that our corpus is not fully representative of the domain.

### Conclusions

We have found sufficient overlap with our derived semantic lexicon to justify the use of the UMLS resources as a starting point for a lexicon for biological language processing (BLP), on the basis of lexical overlap between a lexicon derived from a combination of the UMLS Metathesaurus and the SPECIALIST lexicon, and the terms in a domain corpus. Over 77% of the tokens in the domain corpus are found (through exact match) in the derived lexicon, though only 3% of the unique tokens in the corpus are covered. This shows that the terms captured in the derived lexicon cover the most frequent, and probably the most content-bearing, terms in the domain corpus. Through augmentation with some domain-specific tokenization and named entity extraction, this lexicon can be extremely valuable for BLP.

There remain questions about the utility of the UMLS Semantic Network for BLP. Although we have established a core lexicon for which we have the basic required lexical information — morphosyntactic and semantic information — we have not investigated any potential shortcomings of the UMLS Semantic Network. There are 135 semantic types and 54 relationship types represented in the 2003AC version of the Semantic Network; the number of types is quite small given the complexity of the biomedical domain, and this begs the question of whether it adequately characterizes the semantic distinctions needed for BLP. In contrast, the Gene Ontology resource (Ashburner *et al.*, 2000) contains over 16 000 concepts grouped hierarchically and therefore in principle represents a much more fine-grained semantic breakdown of the domain. The GENIA ontology under development (Ohta *et al.*, 2002) is focused on cell signalling reactions in humans and as such characterizes concepts specific to those processes, again likely to be much more fine-grained than the broad UMLS ontology. The relative utility of different ontologies should be investigated.

Furthermore, we have not explored whether the semantic types associated with terms in our derived lexicon are correct for the specific usages found in our corpus. This is an important issue to be investigated, as a semantic lexicon is only useful to the extent that it captures the appropriate semantics. Finally, we have not assessed the impact of semantic ambiguity on our lexicon — how many of the lexical items are multiply ambiguous, how much of this ambiguity is appropriate to the biological language, and how can we best deal with this ambiguity? These are the questions that we must answer to fully assess the utility of a UMLS-based lexicon for biological language processing.

### Acknowledgement

This work was supported by the Department of Energy under contract W-7405-ENG-36 to the University of California.

### References

Ashburner M, Ball CA, Blake JA, *et al.* 2000. Gene Ontology: tool for the unification of biology. *Nature Genet* **25**(1): 25–29.

Friedman C, Liu H, Shagina L, Johnson S, Hripcsak G. 2001. Evaluating the UMLS as a source of lexical knowledge for medical language processing. *Proc AMIA Symp*, Washington, D.C., 189–193.

Copyright © 2005 John Wiley & Sons, Ltd. 

Hahn U, Romacker M, Schulz S. 1999. How knowledge drives understanding — matching medical ontologies with the needs of medical language processing. *Artif Intell Med* **15**: 25–51.

Johnson SB. 1999. A semantic lexicon for medical language processing. *J Am Med Inform Assoc* **6**(3): 205–218.

McCray AT, Bodenreider O, Malley JD, Browne AC. 2001. Evaluating UMLS strings for natural language processing. *Proc AMIA Symp*, Washington, D.C., 448–452.

Ohta T, Tateisi Y, Mima H, Tsujii J. 2002. GENIA Corpus: an annotated research abstract corpus in molecular biology domain. *Proceedings of the Human Language Technology Conference* (HLT 2002) San Diego, CA; 73–77.

Copyright © 2005 John Wiley & Sons, Ltd.
